# Limited efficacy of enrofloxacin against *Mesomycoplasma ovipneumoniae* infection in domestic lambs

**DOI:** 10.3389/fvets.2026.1944866

**Published:** 2026-10-22

**Authors:** Sobha Sonar, Michael Throolin, Stephen Smith, Jonathon Sago, Eli T. Selong, Noah Adams, Jessica Dibbert, McKenna D. Quirk, Nathan J. Schaff, Helen Schwantje, Christian J. Posbergh, Diane Bimczok

**Affiliations:** 1 Department of Microbiology and Cell Biology, Montana State University, Bozeman, MT, United States; 2 School of Medicine, University of Utah, Salt Lake City, UT, United States; 3 Montana Veterinary Diagnostic Laboratory, Bozeman, MT, United States; 4 Ministry of Water, Land and Resource Stewardship (emerita), Nanaimo, BC, Canada; 5 Department of Animal and Range Sciences, Montana State University, Bozeman, MT, United States

**Keywords:** Baytril, middle ear, *Mycoplasma ovipneumoniae*, respiratory disease, specific-pathogen-free sheep

## Abstract

*Mesomycoplasma ovipneumoniae* is an important respiratory pathogen in sheep that leads to pneumonia and reduced productivity, commonly upon co-infection with other opportunistic microbes. Whether *M. ovipneumoniae* infection can be successfully treated with antibiotics is unclear. Our pilot study evaluated the efficacy of enrofloxacin (Baytril^®^) for treating experimental *M. ovipneumoniae* infection in domestic lambs. Ten 3-month-old specific-pathogen-free lambs were randomly assigned to a treatment and an untreated control group. Lambs were inoculated with fresh nasal wash fluids from *M. ovipneumoniae-*positive sheep. After 4 weeks, the treatment group received enrofloxacin at 0.5 mg/kg in 200 mL of saline intranasally and 5 mg/kg subcutaneously on five consecutive days. Lambs were observed for an additional 4 weeks, with daily health scoring, weekly assessment of weight gain, and weekly nasal swab quantitative (q)PCR analysis for *M. ovipneumoniae* and *Mannheimia haemolytica*. Successful *M. ovipneumoniae* infection of all lambs was confirmed by qPCR 1 week after inoculation. While overall health scores and average daily weight gains were statistically similar between groups, enrofloxacin administration significantly accelerated the rate of body temperature decline over the post-treatment period (*P* = 0.007). Interestingly, all treated lambs tested negative for *M. ovipneumoniae* by qPCR 1 week after treatment. However, there was a re-emergence of *M. ovipneumoniae* infection 2 weeks after treatment with 1/5 lambs testing positive, and 3/5 lambs tested positive 4 weeks after treatment. Co-infection with *Mannheimia haemolytica* was detected in all lambs, with a similar temporary suppression after enrofloxacin treatment. Endpoint tissue swab samples revealed variable qPCR detection of *M. ovipneumoniae*, with a trend for reduced infection of the lower respiratory tract in the treated lambs. However, middle ear swabs from all lambs tested qPCR-positive for *M. ovipneumoniae*, suggesting that the middle ear is a potential reservoir of this pathogen. Tissues collected after euthanasia showed reduced histopathological lung lesions in the enrofloxacin-treated group (Mann–Whitney *U* test, *P* = *0.032*). Amongst four *M. ovipneumoniae* isolates recovered from the lung of one enrofloxacin-treated lamb, one isolate showed reduced susceptibility to enrofloxacin *in vitro*. Overall, our study showed that enrofloxacin treatment was only partially successful in reducing *M. ovipneumoniae* infection in domestic lambs.

## 1 Introduction

*Mesomycoplasma ovipneumoniae* (formerly *Mycoplasma ovipneumoniae)* is a well-known bacterial pathogen that causes asymptomatic to economically significant respiratory infections in *Caprinae* worldwide. The disease, often termed atypical pneumonia, non-progressive pneumonia, or exudative pneumonia, is associated with low mortality and reduced productivity in domestic sheep and goats (1–5). *M. ovipneumoniae* infections have been reported worldwide, although systematic prevalence studies remain rare. In 2011, the USDA's National Animal Health monitoring system conducted its first survey to estimate the prevalence of *M. ovipneumoniae* in domestic sheep in 22 major sheep producing states in United States (6). The study showed that 88% of 453 domestic sheep farms had PCR positive animals and 85% of 408 domestic sheep operations had ELISA-positive animals. In a study conducted in abattoirs in Australia in 2020 and 2021, *M. ovipneumoniae* was detected in 35% of sheep lungs with gross pathological changes consistent with ovine respiratory complex (7). In India, PCR testing identified *M. ovipneumoniae* in 34% of sheep with respiratory disease (8). Prevalence estimates for *M. ovipneumoniae* in China range from 10 to 40%, with higher rates in lambs (9).

The pathogenesis of *M. ovipneumoniae* infection begins by its adherence to the respiratory epithelium, where it is thought to impair ciliary activity, thus affecting mucociliary clearance (10–12). Primary infection with *M. ovipneumoniae* increases host susceptibility to secondary infections with opportunistic pathogens such as *Mannheimia haemolytica, Bibersteinia trehalosi Fusobacterium necrophorum*, or *Pasteurella multocida*, leading to polymicrobial pneumonia with more severe clinical outcomes (13–16).

Domestic sheep (*Ovis aries*) with chronic, often asymptomatic *M. ovipneumoniae* infection act as carriers that can transmit *M. ovipneumoniae* to wild ruminants such as bighorn sheep (*Ovis canadensis*) (15, 17). In bighorn sheep populations, *M. ovipneumoniae* infection with the associated secondary bacterial infections causes severe bacterial pneumonia producing all-age disease outbreaks with mortality ranging from 5 to 100% (18, 19). The issue is widespread in the US with significant impacts on bighorn sheep populations in many states. Therefore, contact with infected domestic *Caprinae* creates a high risk to wild sheep and goat health. Management of disease has been largely limited to physical and temporal separation of potentially infected domestics from wild sheep and goats. Alternatively, removal of chronic *M. ovipneumoniae* carrier domestic sheep is expected to prevent pneumonia in bighorn sheep populations due to a reduced transmission risk (20).

Despite the widespread impacts in domestic and wild sheep populations and the high prevalence of *M. ovipneumoniae* in many of their populations, no effective treatment or preventive strategies are available. While studies have described *in vitro* susceptibility of *M. ovipneumoniae* to antibiotics like gamithromycin, gentamicin, macrolides, tetracycline, and fluoroquinolones, evidence for the successful antibiotic treatment of *M. ovipneumoniae* infection in sheep is very limited (21–24). Use of antibiotics like oxytetracycline, lincomycin, florfenicol, and gamithromycin have resulted in unsuccessful clearance of the *M. ovipneumoniae* infection in domestic sheep (24, 25). One study demonstrated clearance of *M. ovipneumoniae* infection in captive, neonatal bighorn sheep with oral doxycycline, but daily administration over several months was required, which is generally not feasible (23).

We hypothesized that intranasal and subcutaneous administration of enrofloxacin (Baytril^®^) to lambs with *M. ovipneumoniae* infection could eliminate the pathogen from their respiratory tract. Enrofloxacin is a fluoroquinolone antibiotic that is known to have *in vitro* efficacy against *M. ovipneumoniae* with a minimal inhibitory concentration (MIC) range of 0.25 μg/mL to < 0.0625 μg/mL (14, 21, 22). We experimentally infected ten specific-pathogen-free (SPF) lambs with *M. ovipneumoniae* for 4 weeks, then administered the antibiotic and monitored the sheep for another 4 weeks. Our objective was to determine whether enrofloxacin treatment could clear *M. ovipneumoniae* infection in domestic lambs.

## 2 Materials and methods

### 2.1 Study population and animal husbandry

Animal experiments in the current study were approved by the Institutional Animal Care and Use Committee (IACUC) of Montana State University, protocol #2022-158-IA. The lambs were naturally born to mixed breed ewes in the Montana State University specific-pathogen-free (SPF) flock between mid-March to mid-April 2023. The entire SPF flock undergoes testing for *M. ovipneumoniae* and *M. haemolytica* using PCR analysis of nasal swab samples at least once per year, with consistently negative test results far. At approximately 6 weeks of age, all lambs were weaned and provided with *ad libitum* access to water, hay, lamb starter pellets, mineral and selenium supplements. The lambs were vaccinated with a multivalent clostridial vaccine (Bovilis Covexin 8, Merck). For the *M. ovipneumoniae* infection and antibiotic treatment experiment, ten lambs were randomly assigned to two groups and moved to separate animal rooms in the ABSL-2 laboratory at the Johnson Facility Livestock Facility (JFLF). All the lambs in the two experimental groups were kept in heated animal rooms (15.5–16.8 °C) with *ad libitum* access to clean water, mineral, salt and selenium supplements. A standard diet consisting of hay, lamb concentrate, and starter pellets was provided. The animal care personnel responsible for the lambs maintained strict biosecurity measures, ensuring no contact with any other infected animals or non-SPF sheep throughout the experimental period. All personnel changed into sterile scrubs, head and face coverings, boots and double latex gloves prior to entering the animal rooms and showered and changed into new sterile scrubs upon exiting the animal rooms.

### 2.2 Experimental design

Random assignment was performed using R (v 4.3.0) for assigning 10 3-month-old lambs into two experimental groups (Supplementary File 1 Table 1). There were no statistically significant differences in the animal characteristics for sex, litter size, starting weight or age (Table 1). All ten lambs were moved into two separate rooms with five lambs each, with one room housing the enrofloxacin treatment group and the other room housing the control group, 1 week before *M. ovipneumoniae* infection. The husbandry staff, undergoing all the biosafety measures, always entered and handled the enrofloxacin-treated group before entering the room with untreated controls.

**Table 1:** Characteristics of experimental lambs.

Experimental group	*N*	Treatment method	Males	Litter size	Weight at start (kg)^b^	Age at start (d)^b^
Control	5	None	2	2.6 ± 0.5	35.2 ± 5.0	91.6 ± 0.9
Enrofloxacin treatment^a^	5	Nasal flush and Subcutaneous^a^	2	3.0 ± 0.7	36.2 ± 2.5	92.2 ± 0.8
*P* value			>0.999	0.643	0.989	0.429

^a^Treatment with antibiotic enrofloxacin after 4 weeks of infection with Nasal wash and Subcutaneous route. ^b^Start day refers to the day of infection. Groups were compared using Fisher's exact test (for sex distribution) or the Mann–Whitney *U* test (other parameters). Normality tests were performed using a Shapiro–Wilk test, and data did not follow a Gaussian distribution.

### *2.3 M. ovipneumoniae* infection with fresh nasal wash inocula

Nasal wash collection was performed under animal protocol 2023-296-AA as described previously (24, 26). Eighteen domestic sheep at Montana State University's Red Bluff Research Ranch were selected for inoculum collection based on a recent positive *M. ovipneumoniae* qPCR test from nasal swab samples. 17/18 lambs also tested positive for *M. haemolytica* by qPCR. *M. haemolytica* isolates from two animals underwent antimicrobial susceptibility testing at the Montana Veterinary Diagnostic Laboratory (MVDL) and were found susceptible to ceftiofur. For preparation of the bacterial inoculum, these sheep were subjected to nasal washes by squirting 30 ml of sterile PBS into each sheep nostril and collecting the flush fluid into clean, single polyethylene bags (ZipLock^®^, S.C. Johnson and Son, Racine, Wisconsin, USA). The nasal wash fluid was filtered through a 70 μm cell strainer, pooled and diluted with tryptic soy broth in ratio 1:1. After dilution, the wash fluid was incubated with the antibiotic ceftiofur (100 μg/mL, MWI Animal Health, Boise, ID) for 2 h at 37 °C to eliminate *M. haemolytica* and other *Pasteurellaceae* and then concentrated by centrifugation at 10,000*g* for 30 min. The ceftiofur-treated nasal wash was streaked on blood agar, and no growth of *M. haemolytica* was detected following the ceftiofur treatment. The final inoculum was prepared by resuspending the pellets in sterile Phosphate Buffer Saline (PBS) and making 50 mL aliquots. Infection was achieved by infusing 15 mL of inoculum into each naris, 10 mL orally, and 5 mL into each conjunctival sac of each lamb.

### 2.4 Health scoring and clinical assessment

Lambs subjected to experimental infection were closely observed by trained personnel at the Johnson Family Livestock Facility, for clinical changes throughout the study period. The total health score or disease severity was determined twice daily using a clinical scoring table previously used in Jacobson et al. (26) (Supplementary File 1 Table 2) based on respiratory signs, behavioral condition, appetite, and non-experimental medications. Biweekly rectal temperatures were measured and additionally recorded whenever clinical illness was suspected. Body weight was monitored on a weekly basis.

### 2.5 Antibiotic treatment

Enrofloxacin (Baytril^®^ 100, 100 mg/mL Antimicrobial Injectable Solution, Elanco Animal Health, Indianapolis, Indiana, USA) was administered to the lambs in the treatment group both intranasally and subcutaneously, every 24 h for 5 days. For intranasal administration, enrofloxacin was diluted to 0.5 mg/kg in 200 mL of lukewarm saline in a nasal wash bottle (Waterpulse^®^ Neti Pot, 300 mL, Industrial Co., Ltd., Shanghai, China) just before administration. The lambs were restrained and held with the neck in ventroflexion, angling the nasal passage at ~90-degree angle to the ground to avoid aspiration into the lungs. The tip of the nasal wash bottle was placed into one of the nostrils and manual pressure was applied to the bottle to squeeze about 100 mL of the treatment solution first into the right and after 60 s into the left nostril. For parenteral administration, 5 mg/kg of enrofloxacin was administered via subcutaneous injection. A new injection site was used every day to avoid injection-site trauma.

### 2.6 Detection of *M. ovipneumoniae* and *M. haemolytica*

Nasal swab samples for the detection of *M. ovipneumoniae* and *M. haemolytica* were collected weekly using flocked 15-cm polyester swabs (Puritan Flocked 25-3317-U BT) in 1 mL of transport media (85% Dulbecco's modified Eagle medium and 15% glycerol) placed in 15 mL Falcon tubes on ice for transport. The samples were then pulsed vortexed for 30 s prior to removing the nasal swab from the tube and additional 1 mL of transport media was added to the graduated tube. One mL of this swab sample was transferred each to a cryovial and store at −80 °C. The epMotion 5075 (Eppendorf, CT, USA) automated liquid handling system was used for DNA extraction from the swab samples using the Mag-Bind^®^ Viral DNA/RNA 96 Kit (Omega Bio-Tek, Norcross, Georgia, USA) following the manufacturer's protocol. All extraction plates included negative extraction control samples containing Nuclease Free Water (Promega Inc., Madison, Wisconsin, USA) and positive extraction controls from known positive samples, which were included as negative/positive extraction controls in the corresponding PCR assays. Lastly, the extracted DNA was stored at −20 °C for further use.

The qPCR assays for *M. ovipneumoniae* and *M. haemolytica* quantification were performed in our laboratory. The master mix for the qPCR assay consisted of 12.5 μL of iTaq universal SYBR green supermix (Bio-Rad, Hercules, CA, USA), 2.5 μL of premixed primers for the *M. ovipneumoniae* p113 gene: forward: 5′-GGG GTG CGC AAC ATT AGT TA-3′ and reverse: 5′-CTT ACT GCT GCC TCC CGT AG-3′ (27), Prime PCR Customs (Bio-Rad, Hercules, CA, USA), 1.4 μL of DNA template and 8.6 μL of nuclease free water (Promega Inc., Madison, Wisconsin, USA). Amplification occurred under following conditions: initiation step of 5 min at 95 °C, followed by 40 cycles of 95 °C denaturation for 15 s, 60 °C annealing for 30 s and 60 °C elongation for 30 s using a MicroAmp^TM^ Enduraplate^TM^ Optical 96-Well fast Clear Reaction Plates (Applied Biosystems, Thermo Fisher Scientific, Waltman, MA, USA). QuantStudio^TM^ Design and Analysis Software 5 (Thermo Fisher Scientific, Walthman, MA) were used to determine the quantification cycles (*C*_t_) threshold and for melt curve analysis.

The master mix for the *M. haemolytica* qPCR assay consisted of 10 μL of the iTaq universal SYBR green supermix (Bio-Rad, Hercules, CA, USA), 1 μL of both forward and reverse primers for the *M. haemolytica* hemolysin D gene, designed from GenBank sequence GCF_002285575.1 with forward: 5′-GCC AAG TGG TGT ATT GCT CC-3′ and reverse: 5'-AGA GTC CTT GCC GGT GAT TG-3′(Eurofins primers, KY, USA), 1 μL of DNA template and 7 μL of nuclease free water (Promega Inc., Madison, Wisconsin, USA) to reach a final volume of 20 μL. Amplification was performed using MicroAmp^TM^ Enduraplate^TM^ Optical 96-Well Fast Clear Reaction Plates (Applied Biosystems, Thermo Fisher Scientific, Waltman, MA, USA) under the following conditions: initiation for 5 min at 95 C, 40 cycles of 95 °C denaturation for 15 s, 60 °C annealing for 30 s and 60 °C elongation for 30 s. All reactions plates included negative controls consisting of reaction mixture with nuclease free water instead of the template DNA and another having a negative extraction control. QuantStudio^TM^ Design and Analysis Software 5 (Thermo Fisher Scientific, Walthman, MA) was used to determine the quantification cycle (*C*_t_) threshold and for melt curve analysis.

To construct a standard curve for DNA quantification and to determine the limit of detection of the assay, *M. ovipneumoniae* standard type strain Y98 (ATCC 29419™, NCTC 10151) was used and seven 1 in 10 dilutions with a range of 10^0^ to 10^6^ cells/mL were prepared. For the required cell concentration, a ZE5™ Cytometer (Bio-Rad, Hercules, CA, USA) was used following the flow cytometry protocol described by Jacobson et al. (26). For *M. haemolytica* standard type strain Newsom and Cross (serotype 2, ATCC 33396) was used, and seven 1 in 10 dilutions with a range of 10^0^ to 10^6^ CFUs/mL were prepared. The DNA was extracted from these dilutions as described above. Five replicates of each DNA sample were analyzed in a Quant Studio 5 Real Time PCR instrument (Applied Biosystems, Thermo Fisher Scientific, Waltman, MA, USA) using the PCR protocol described above, respectively, which confirmed an efficiency of 85.8% for the p113 gene detection for *M. ovipneumoniae* and an efficiency of 95.4% for the hemolysin D gene for *M. haemolytica* (Supplementary File 2). All PCR tests included positive and negative template controls and positive and negative extraction controls for quality control.

### 2.7 Euthanasia and sample collection

Four weeks after initiation of enrofloxacin treatment, all experimental lambs were sedated with xylazine (Rompun^®^, Dechra Pharmaceuticals, Boston, Massachusetts, USA, 100 mg/mL) and subsequently euthanized by intravenous administration of pentobarbital sodium (Fatal plus^®^, Vortech Pharmaceuticals, Dearborn, Michigan, USA) at a dose rate of 1 ml per 4.5 kg plus 10% via jugular venipuncture. Lambs were then transferred to the MVDL within 30 min of euthanasia for necropsy and sample collection. Swab samples were collected from the paranasal sinus, trachea, bronchi, caudal nasal cavities, and middle ear and were processed as described above. Swab samples from the left and right nasal cavity were pooled together, as were swabs from both middle ears.

### 2.8 Pathological and histological evaluation

The upper and lower respiratory tract and the pleural cavity were evaluated for gross pathology by two trained veterinary pathologists (SSm and JS). For histopathological evaluation, lung samples were fixed in formalin, followed by paraffin embedding, and then were subjected to hematoxylin and eosin (H-E) staining at the MVDL. An Olympus BH-2 3 Head Microscope (Olympus, Feasterville, PA, USA) was used to examine and score the histopathological changes based on criteria described in Jacobson et al. (26). The examiner was blinded to the experimental group. Transmission electron microscopy (TEM) was performed to investigate the ciliary structure of the trachea from two treated lambs (7222DB and 7920DB) and two untreated control lambs (8116DB and 8518DB). A small portion of tracheal tissue was fixed in 3% glutaraldehyde and processed as previously described (28). Samples were visualized on Tecnai Spirit Bio-Twin Transmission Electron Microscope (FEI, Hillsboro, OR, USA).

### 2.9 Cultivation of *M. ovipneumoniae* and *M. haemolytica* from respiratory tract samples

Frozen lung tissue samples collected in cryovial tubes and stored in −80 °C from lambs in both the treated and untreated control group were used to isolate *M. ovipneumoniae* following the protocol from Daee et al. (29) with slight modifications. Lung tissues from six different lambs, two from the treated groups (7122DB and 8216DB) and four from untreated control lambs (7022DB, 7414DB, 8020DB, and 8116DB) were placed in the 15 mL tubes with 3 mL of Mycoplasma media (Supplementary File 1 Table 3) and kept at 4 °C for 48 h. The samples were then vortexed for 60 s and 100 μL of the sample was inoculated over the Mycoplasma agar media using L-spreader and incubated at 37 °C. The plates were examined daily for transparent, flat and fried egg like colonies using a stereomicroscope for 14 days. Single colonies with characteristic morphology were picked using an inoculating loop and transferred to 100 μL of Mycoplasma media and incubated at 37 °C for an additional 3 to 14 days until a color change was visible. The inoculated media were examined daily for the color change from red to yellow, indicating metabolic changes due to growth of *M. ovipneumoniae*. Eleven isolates were recovered, four from 8216DB (treated), five from 7022DB, one from 7414DB, and one from 8020DB (all untreated). Isolates were expanded to sufficient volumes by transferring into new media multiple times and then were frozen for later MIC analysis.

For the isolation of *M. haemolytica*, 100 μL of frozen nasal swab samples from both the treated and the untreated control groups were inoculated onto blood agar plates and incubated overnight at 37 °C. Colonies characteristic of *M. haemolytica* forming small, gray, beta-hemolysis were transferred onto new blood agar plates for secondary culture. Biochemical tests for indole, catalase, oxidase, gram staining and growth on MacConkey agar plates were performed to confirm the identity of the *M. haemolytica* colonies. The colonies were then transferred to brain heart infusion (BHI) broth media and cultured for 18–24 h at 37 °C in an incubator shaker at 250 rpm. A total of five *M. haemolytica* isolates were recovered, two from 8216DB, and one each from 7122DB, 8020DB, and 8518DB. DNA extraction for all the broth cultures was performed, and qPCR analysis was conducted to detect the *M. ovipneumoniae* p113 and the *M. haemolytica* hemolysin D gene in these isolates to confirm bacterial identity.

### 2.10 Antibiotic susceptibility testing for both the laboratory and clinical isolates

For the antibiotic susceptibility testing of the clinical isolates of *M. ovipneumoniae*, a broth microdilution assay (30) was performed as previously described (24). MIC assays were performed using frozen aliquots of the recovered isolates that had been transferred to new media 5–12 times to achieve sufficient volumes. Enrofloxacin concentrations from 0.0006 to 640 μg/mL were inoculated with the isolates from the lung tissues and the standard type strain Y98 ATCC 29419 in a 96-well plate and incubated at 37 °C. The plates were examined every 24 h for 14 days to detect any color change in the culture depicting acid production and bacterial growth. The lowest dilution without a color change was recorded as the minimum inhibitory concentration (MIC) against *M. ovipneumoniae*.

The antibiotic broth microdilution assay for *M. haemolytica* was performed using the Clinical and Laboratory Standard Institute guidelines (31). Enrofloxacin concentrations from 0.031 to 32 μg/mL were tested against the nasal swab isolates and the standard type strain Newsom and Cross (serotype 2, ATCC 33396) in a 96-well plate and incubated overnight at 37 °C in an incubator shaker. For *M. haemolytica*, the lowest dilution with no growth, based on optical density readings at 600 nm, was defined as the MIC. All bacterial isolates were subcultured between five and twelve times prior to the antibiotic susceptibility testing.

### 2.11 Statistical analysis

Data were analyzed using GraphPad Prism (v10) or R Statistical Software (v4.5.2) (32) and are shown as mean ± SD unless otherwise specified.

Data for the total health scores, temperature, and average daily weight gains (ADG) models were processed with the tidyverse package (v2.0.0) (33), the linear mixed models were run with the lme4 package (v1.1.38) (34) for continuous outcomes (temperature and ADG), while a zero-inflated Poisson generalized linear mixed-model was run for discrete outcomes (Total Health Score) with the glmm TMB package (v1.1.14) (35). Plots related to these models were generated with the ggplot2 package (v4.0.1) (36) and ggpubr package (v0.6.3) (37). For these models, the primary treatment of interest was antibiotic administration at Week 0. Consequently, all analyses were restricted to the post-treatment period (Week ≥0). This restriction isolates the therapeutic effect from the earlier infection dynamics that occurred between Week −4 and Week 0, providing a cleaner assessment of treatment efficacy.

All models included treatment (enrofloxacin or untreated control) and an interaction term involving Time (Week). Due to the hierarchical structure of the data, specifically, repeated measures taken on the same lamb over time and the initial clustering of lambs born to the same ewe, we evaluated nested random effects. However, due to singular fit constraints associated with the limited number of ewe clusters, the ewe-level random effect was omitted to prevent overparameterization. Consequently, to account for the non-independence of repeated measures taken on the same animal, all models included a random intercept only for Lamb ID, where $v_{lamb}\sim N\left(0,\sigma_{lamb}^{2}\;\right)$.

#### 2.11.1 Body temperature and average daily weight gain (ADG) models

For the continuous outcomes *Y*_*ij*_ (temperature and ADG) for the *i*^*th*^ lamb at the *j*^*th*^ week, the LMM is defined as:


$$\begin{array}{l} Y_{ij}=\beta_{0}+\beta_{1}Treatment_{i}+\beta_{2}Week_{j}+{\beta_{3}Treatment}_{i}\times Week_{j}\\ +v_{i}+\epsilon_{ij} \end{array}$$

Where: β_0_: overall intercept. β_1_, β_2_: fixed effect coefficients for Treatment and Week. β_3_: the coefficient for the interaction term between Treatment and Week. ν_*i*_: a term accounting for repeated measures on the same lamb, $\nu_{i}\sim N\left(0,\sigma_{lamb}^{2}\right)$, ϵ_*ij*_: the residual error, $\epsilon_{ij}\sim N\left(0,\sigma_{residual}^{2}\;\right)$.

#### 2.11.2 Total health score model

For the discrete outcome Total Health Score, data was analyzed using a zero-inflated Poisson generalized linear mixed-effects model fitted with the glmm TMB package in R. This modeling framework accounts for both the count nature of the outcome and the hierarchical structure of the data, including repeated measures within lambs and clustering of lambs within ewes.

*Y*_*ij*_ denotes the total health score for the *i*th lamb at the *j*th week during the post-treatment period. The outcome was modeled using a zero-inflated Poisson distribution:


$$\begin{array}{l} Y_{ij}\sim ZIP\left(\lambda_{ij},\pi \;\right) \end{array}$$

The probability mass function can be written as:


$$\mathrm{Pr}(Y_{ij}=y\;|v_{i})=\{\begin{array}{c} \pi +(1-\pi )e^{-\lambda_{ij}},\;\;\;\text{for}\;y\;=0\;\\ (1-\pi )\frac{\lambda_{ij}^{y}e^{-\lambda_{ij}}}{y!},\text{for}\;y\;=1,2,\ldots \end{array}$$

The zero-inflation probability was modeled with a logistic link function and was intercept only:


$$\begin{array}{l} \text{logit}\left(\pi \right)\;=\;\gamma_{0} \end{array}$$

The conditional mean of the Poisson component was modeled using a log link:


$$\begin{array}{l} \log\left(\lambda_{ij}\right)=\beta_{0}+\beta_{1}Treatment_{i}+\beta_{2}Week_{j}+{\beta_{3}Treatment}_{i}\\ \times Week_{j}+v_{i} \end{array}$$

Where the random effect follows an independent normal distribution:


$$\begin{array}{l} \nu_{i}\sim N\left(0,\sigma_{lamb}^{2}\right) \end{array}$$

We employed two distinct model structures to answer separate clinical questions regarding the impact of the treatment. First, the longitudinal trajectory analysis which allows the model to capture non-linear recovery trends without assuming a fixed, straight-line relationship over time in the post treatment phase. This analysis determines how the treatment's effect on the outcome was modified by time over the 4-week recovery period (Weeks 0–4) The primary test was the Treatment × Time interaction. Secondly, an endpoint analysis was performed to compare the change in outcome in the treated group to the change in the control group by analyzing the observations only at the Week 0 and Week 4 post treatment. This analysis allows us to determine if the outcome at the study endpoint (Week 4) differed significantly from the baseline (Week 0) between the treatment groups. Data were restricted to only observations at Week 0 and Week 4, and time was treated as a categorical factor (0 *or* 4). The primary test was the Treatment × Time interaction, which compares the change in outcome in the treated group to the change in the control group.

#### 2.11.3 Model comparison and statistical inference

The significance of the primary effects (the Treatment × Time interaction for both analyses) was determined using a Likelihood Ratio Test (LRT). This involved comparing two nested models: the full model (containing the interaction) against the reduced model (containing only main effects).


$$\begin{array}{l} LRT\;Statistic=-2\left(\log L_{Reduced}-\log L_{Full}\right)\sim \chi_{df}^{2} \end{array}$$

The LRT generates a Chi-square (χ^2^) statistic and its associated *p*-value for the interaction term, with degrees of freedom (df) equal to the difference in the number of fixed effect parameters between the two models. A *P* value less than 0.05 was considered statistically significant.

## 3 Results

### 3.1 Colonization of the upper respiratory tract with *M. ovipneumoniae* and *M. haemolytica* following experimental inoculation of specific pathogen free lambs with ceftiofur-treated nasal wash samples

Ten 3-month-old specific-pathogen-free lambs were randomly assigned to two groups, an enrofloxacin treatment and an untreated control group. Lambs were inoculated with fresh nasal wash fluids from *M. ovipneumoniae-*positive sheep. After 4 weeks, the treatment group received intranasal and systemic enrofloxacin whereas the other remained untreated (Figure 1A). An overview of the results for each animal is provided in Supplementary File 1 Table 1. Similar to our previous studies (24, 26), inoculation of SPF-lambs with ceftiofur-treated nasal wash samples from *M. ovipneumoniae*-positive sheep resulted in colonization of the nasal mucosa with *M. ovipneumoniae* 1 week after inoculation, as detected by qPCR analysis of nasal swabs (Figure 1B). Despite the *in vitro* treatment and susceptibility of *M. haemolytica* to ceftiofur as confirmed by susceptibility testing performed by the MVDL, qPCR analysis demonstrated *M. haemolytica* colonization in the upper respiratory tract of 9/10 lambs at 2 weeks post-infection (week −2; Figure 1C), indicating secondary infection after *M. ovipneumoniae*. All ten infected specific-pathogen-free lambs tested positive for both *M. ovipneumoniae* and *M. haemolytica* at the time when enrofloxacin was administered.

**Figure 1:**
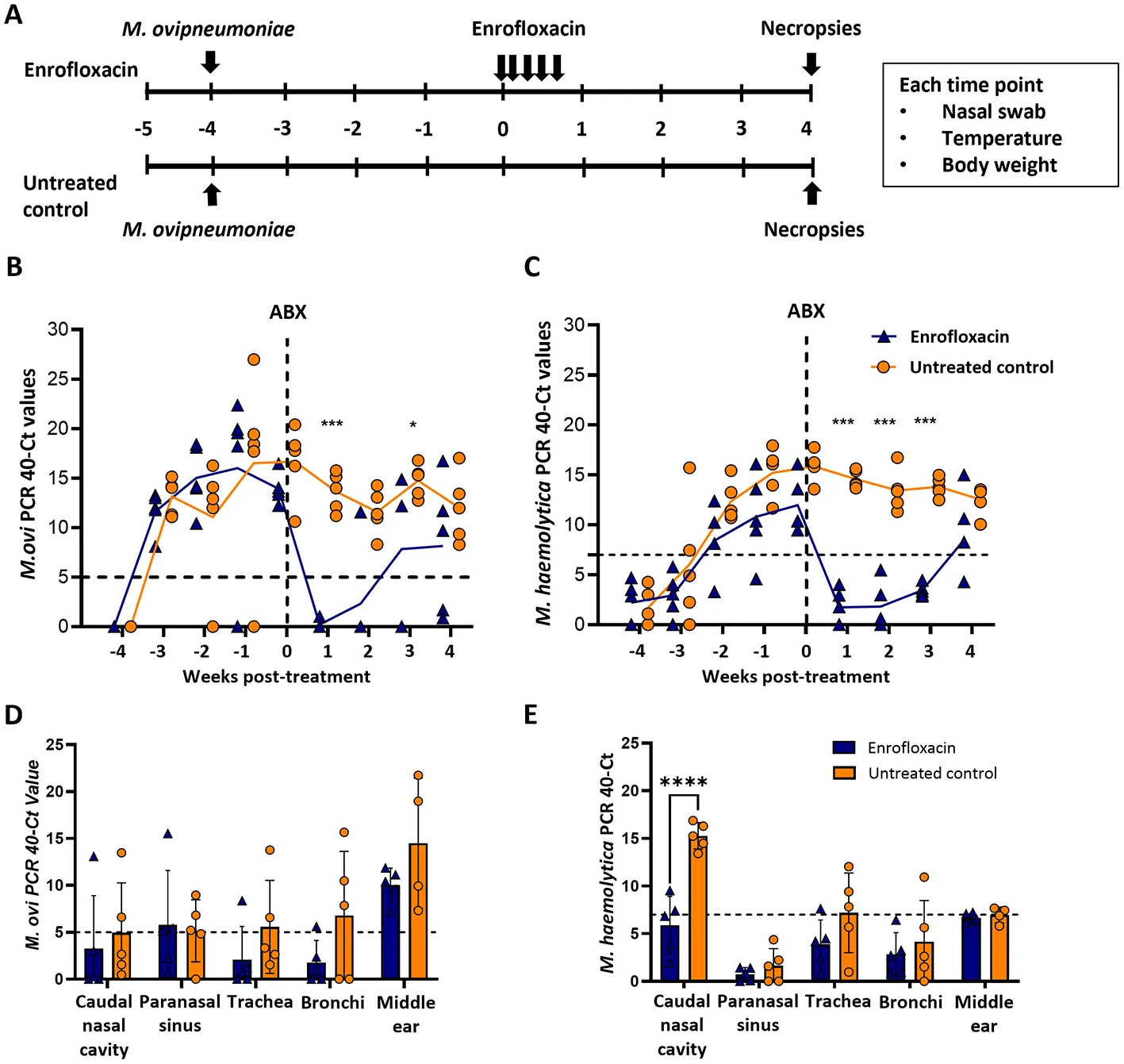
Enrofloxacin resulted in temporary suppression of *M. ovipneumoniae* and secondary *M. haemolytica* infection in the upper respiratory tract but persistent infection of the middle ear. **(A)** Experimental design. **(B, C)** *M. ovipneumoniae* and *M. haemolytica* detection in nasal swabs using qPCR. Data are shown as 40 minus Ct values. Purple triangles: Enrofloxacin (*n* = 5). Orange circles: untreated controls (*n* = 5). Two-way ANOVA analysis with Sidak's multiple comparison test shows significant differences between the groups, ***P < 0.001 and *P < 0.05. **(D, E)** qPCR analysis of *M. ovipneumoniae* and *M. haemolytica* infection displayed as 40-Ct values in swabs collected from different postmortem sites. Two-way ANOVA analysis with Sidak's multiple comparison test, ****P < 0.0001. Dashed lines indicate limits of detection.

### 3.2 Enrofloxacin led to temporary suppression of *M. ovipneumoniae* and *M. haemolytica* in the nasal cavity of experimentally infected lambs

PCR testing of nasal swabs for *M. ovipneumoniae* and *M. haemolytica* continued after enrofloxacin administration. Notably, all five lambs in the enrofloxacin treated group tested *M. ovipneumoniae* negative 1 week after receiving the first enrofloxacin dose. However, 1/5 of enrofloxacin-treated lambs (20%) tested *M. ovipneumoniae* positive at week 2, 2/5 (40%) tested positive at week 3, and 3/5 (60%) tested positive at week 4 after treatment. All *M. ovipneumoniae-*infected lambs in the untreated control group tested *M. ovipneumoniae* positive throughout the entire experiment (Figure 1B).

Likewise, all treated lambs tested *M. haemolytica* negative 1 week after enrofloxacin administration (Figure 1C). However, as with *M. ovipneumoniae*, there was a re-emergence of *M. haemolytica* infection in the fourth week after treatment (experimental end point) with the same 3/5 *M. ovipneumoniae* positive lambs (7122DB, 7625DB, 8216DB) also testing *M. haemolytica* positive. All untreated lambs remained *M. haemolytica* positive throughout the entire study.

### 3.3 Impact of enrofloxacin treatment on the colonization of different respiratory tissue sites with *M. ovipneumoniae* and *M. haemolytica*

At the experimental endpoint 8 weeks after inoculation and 4 weeks post treatment initiation, all lambs were euthanized, and additional swab samples were collected from the caudal nasal cavities, paranasal sinus, trachea, bronchi, and the middle ear. qPCR analysis revealed inconsistent recovery of *M. ovipneumoniae* from these sites (Figure 1D). While pathogen loads in the enrofloxacin group trended lower, there was no significant difference in the colonization of *M. ovipneumoniae* in the upper and the lower respiratory tracts between enrofloxacin-treated and untreated control lambs (Figure 1D), with majority of swabs from both groups testing negative. Interestingly, high levels of *M. haemolytica* were detected in the caudal nasal cavity of untreated lambs at the experimental endpoint, whereas the colonization in the other respiratory sites was inconsistent (Figure 1E). Importantly, enrofloxacin-treatment was associated with a significant decrease (2-way ANOVA, *P* ≤ 0.0001) in *M. haemolytica* in the caudal nasal cavity (Figure 1E). While nasal swab samples of 2/5 of the enrofloxacin lambs remained negative for *M. ovipneumoniae* and *M. haemolytica* (Figures 1B, C), all swab samples collected from the middle ear of both enrofloxacin-treated and untreated lambs tested PCR-positive for *M. ovipneumoniae* but PCR-negative for *M. haemolytica* (Figures 1D, E).

### 3.4 Enrofloxacin treatment led to a moderate decrease in lung histopathology but no difference in gross pathological lesions

At necropsy, lungs were inspected for visible gross lesions (Figures 2A, B). Overall, only minor, inconsistent pathological abnormalities were observed with no apparent differences between the groups. Lamb 7625DB from treated and 7022DB from untreated control group have mildly enlarged retropharyngeal lymph nodes (Figures 2C, D). One lamb in the treatment group, 7920DB, had a fibrous adhesion between the right cranial lobe and the adjacent body wall (Figure 2C). Similarly, one lamb in the untreated control group, 7414DB, had some focal pleural adhesions and consolidations in the right and left cranial and the right middle lobes (Figures 2B, D). 8116DB from the untreated control group had consolidated middle and cranial lung lobes. Additionally, 8216DB from the treated group had a hemorrhagic right cranial lobe. 2/5 lambs in both groups, 7122DB and 7222DB (treated), and 8020DB and 8516DB (untreated control) did not have any gross pathological lesions (Figures 2C, D). These findings suggest that the lambs in the two groups exhibited similar gross lesions in their lungs.

**Figure 2:**
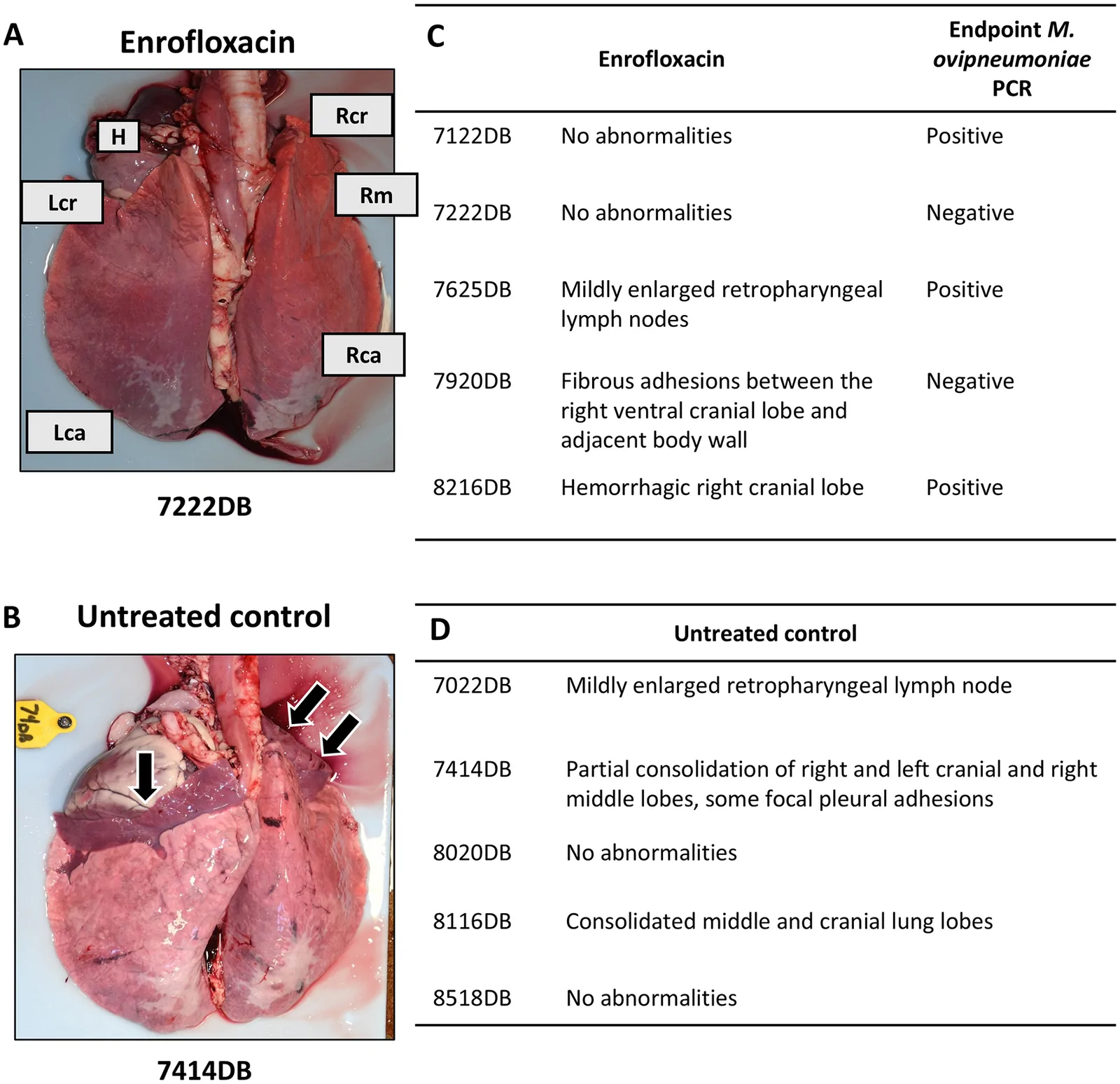
Analysis of gross pathological lesions in the lungs of enrofloxacin-treated lambs compared to the untreated controls. **(A,B)** Representative lungs from an enrofloxacin treated and an untreated control lamb at 8 weeks post *M. ovipneumoniae* infection. H, heart; Lcr, left cranial lung; Rcr, right cranial lung; Lca, left caudal lung; Rca, right caudal lung; Rm, right middle lobe. An untreated control lung showed partially consolidated middle and cranial lung lobes (see arrows). **(C, D)** Gross pathological findings from each individual lamb at necropsy. **(C)** Endpoint *M. ovipneumoniae* detection refers to a positive qPCR result in a nasal swab sample collected at the experimental end point as shown in Figure 1B.

We performed blinded histopathological analysis of respiratory tract tissues and scored the samples according to Jacobson et al. (26). The majority of tissue samples had no or only moderate changes (Figures 3A–F). In some untreated control lambs, peribronchiolar cuffing, neutrophil accumulation in the bronchioles, and development of lymphoid follicles (BALT) were detected. We found a trend for decreased inflammation in both the bronchiolar areas and the alveolar space and decreased BALT hyperplasia in the enrofloxacin-treated lambs compared to the untreated controls. Statistical analysis showed a small but significant decrease in the pulmonary edema and congestion score (Mann–Whitney *U* test, *P* = 0.040) and in the combined histological lung score (Mann–Whitney *U* test, *P* = 0.032) for the enrofloxacin-treated compared with the untreated control lambs (Table 2).

**Figure 3:**
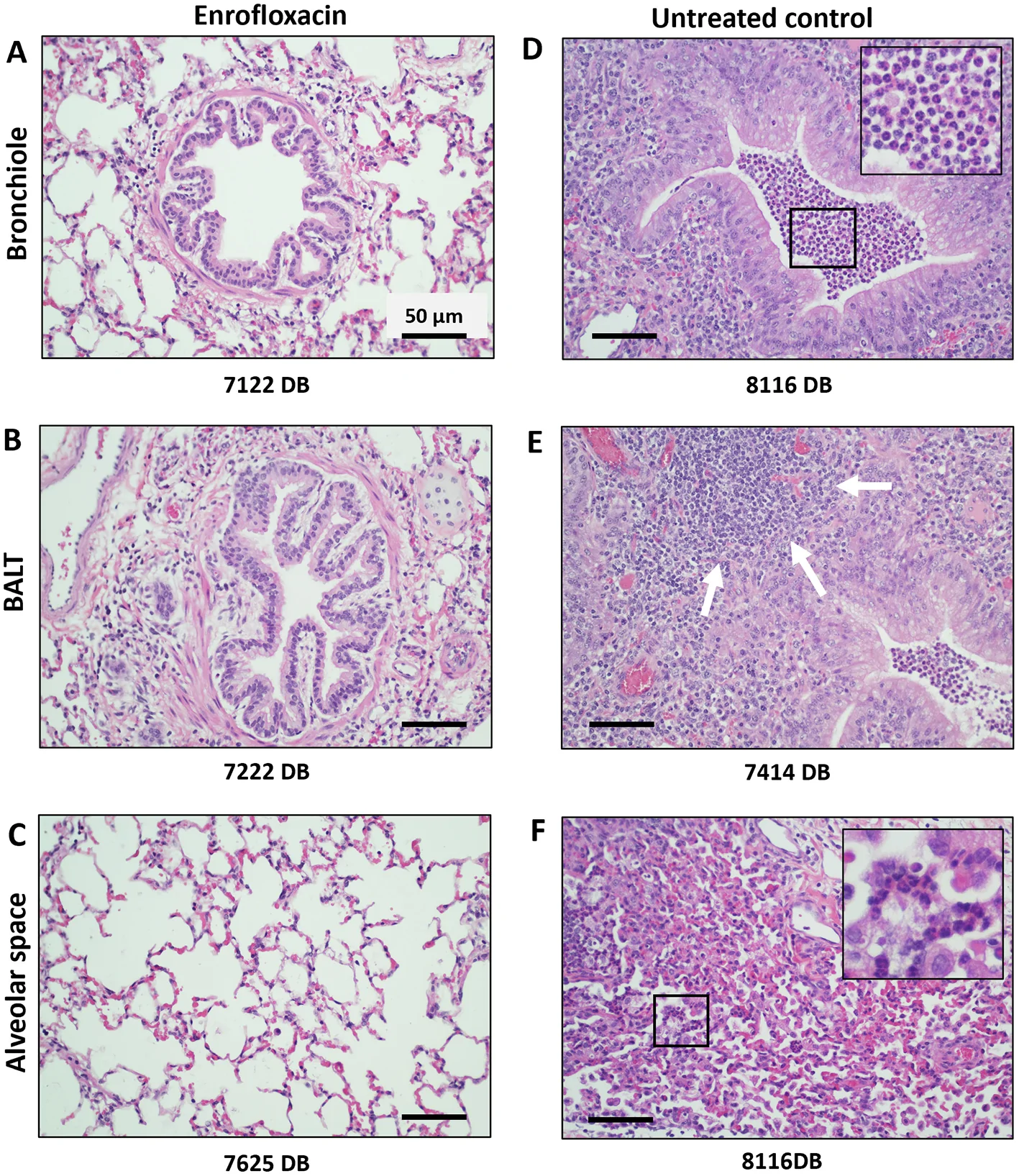
Enrofloxacin treatment led to decreased histopathological lung lesions. **(A)** Bronchioles from enrofloxacin-treated lamb 7122DB. **(B)** Bronchiole without Bronchus Associated Lymphoid Tissue (BALT) in the enrofloxacin-treated lamb 7222DB. **(C)** Normal alveolar space in the enrofloxacin-treated lamb 7625DB. **(D)** Infiltration of immune cells in the lumen and surrounding a bronchiole of a representative untreated control lamb 8116DB. Insert indicates the accumulation of neutrophils in the bronchiolar lumen (**E)** Bronchiole with Bronchus Associated Lymphoid Tissue (BALT) follicle hyperplasia in the untreated control 7414DB. **(F)** Tissue congestion and infiltration of alveolar spaces with inflammatory cells, neutrophils and lymphoid cells in the untreated control lamb 8116DB (see inset). All slides were stained with hematoxylin-eosin, bar = 50 μm.

**Table 2:** Lung histopathological scoring.

Histopathological scoring parameters	Enrofloxacin treatment (*n* = 5)	Untreated control (*n* = 5)	*P-*value
Pulmonary edema and congestion	0.8 ± 0.6	1.7 ± 0.4	0.040^*^
Alveolar and interstitial inflammation	0.0 ± 0.0	0.8 ± 0.9	0.167
Bronchiolar inflammation	0.0 ± 0.0	0.6 ± 0.8	0.167
BALT hyperplasia	0.1 ± 0.1	0.7 ± 0.8	0.286
Total score	1.1 ± 0.4	3.8 ± 2.1	0.032^*^

Groups were analyzed using the Mann–Whitney *U* test. ^*^Denotes significant differences between groups at *P* < 0.01. The total score represents the sum of the four individual score calculated for each animal.

### 3.5 Transmission electron microscopy reveals reduced ciliary damage in the trachea of enrofloxacin treated lambs

To visualize the effect of the *M. ovipneumoniae* infection and enrofloxacin treatment on ciliary morphology of the tracheal epithelium, we also performed transmission electron microscopy (TEM) of tracheal sections from two lambs per group (Figure 4), the enrofloxacin-treated lambs 7222DB and 7920DB that remained PCR-negative up to 4 weeks after treatment, and 8116DB and 8518DB from the untreated control group. The TEM images revealed the disruption of cilia present at the epithelial cell membrane in the trachea of the two untreated control lambs. In contrast, intact cilia with a characteristic microtubule core were observed in the trachea of the two enrofloxacin-treated lambs that were analyzed.

**Figure 4:**
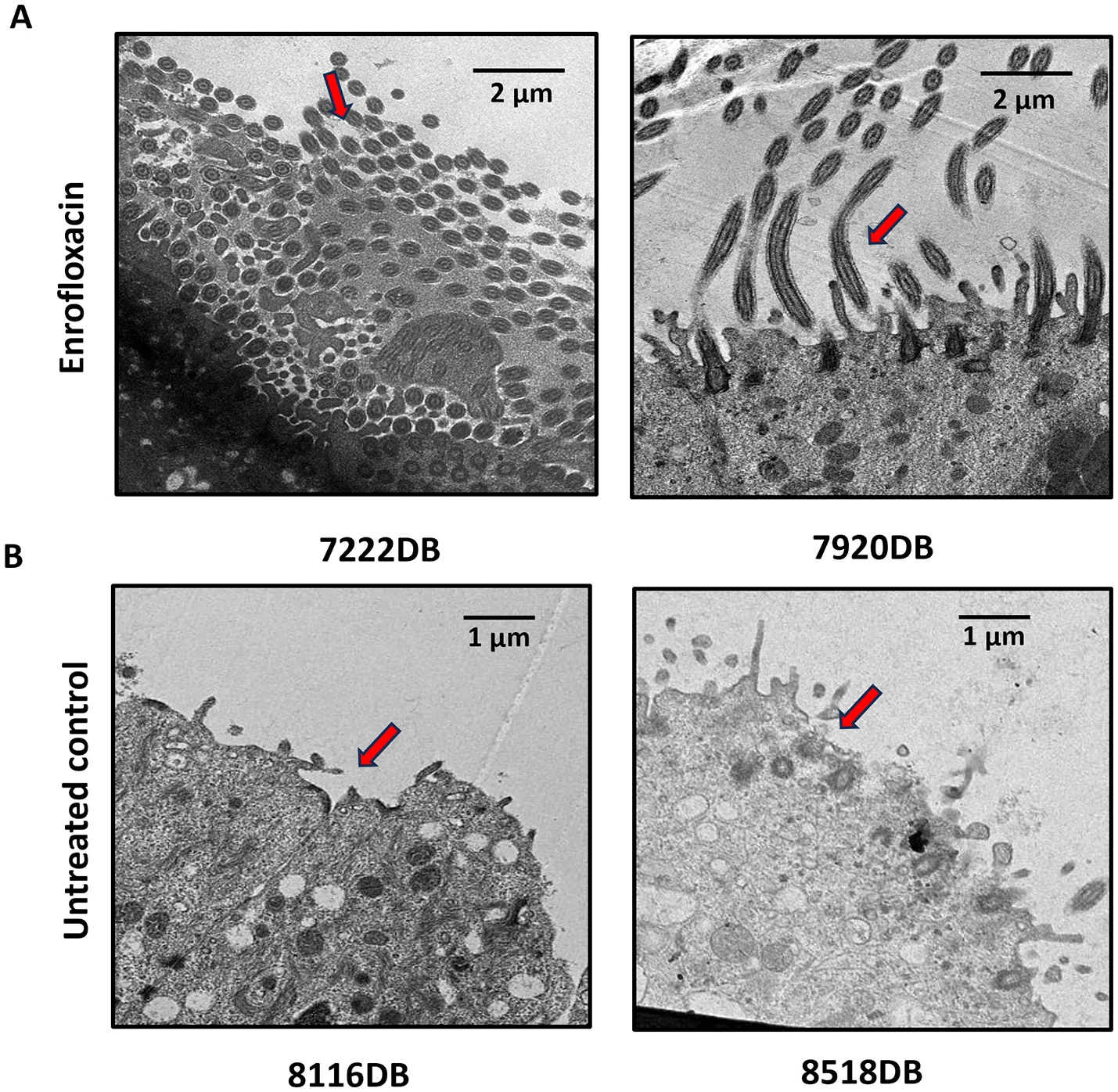
Transmission electron microscopy (TEM) images of reduced ciliary projections in the tracheal mucosa of untreated lambs but not enrofloxacin treated lambs. **(A)** Transmission electron microscopy shows cilia projecting from respiratory epithelial cells in ultrathin sections of trachea from *M. ovipneumoniae* infected and enrofloxacin treated lambs 7222DB and 7920DB. Scale bar 2 μm. Arrows depict normal cilia in the trachea. **(B)** Images showing respiratory epithelial cell surfaces in ultrathin sections of trachea from *M. ovipneumoniae* infected but untreated control lambs 8116DB and 8518DB. Scale bar 1 μm. Arrows depict reduced cilia and disrupted tracheal epithelium. The lamb ID is shown at the bottom of each image.

### 3.6 Some *M. ovipneumoniae* and *M. haemolytica* isolates from the enrofloxacin treated group exhibited reduced antimicrobial susceptibility

To determine whether the reemergence of the *M. ovipneumoniae* and *M. haemolytica* in some of the lambs after treatment and initial successful elimination of the bacteria from the nasal cavity was due to the development of antimicrobial resistance, we performed antimicrobial susceptibility assays of *M. ovipneumoniae* isolated from lung tissue at the experimental endpoint. We successfully cultivated eleven *M. ovipneumoniae* isolates from the lung tissues of one enrofloxacin-treated (*n* = 4 isolates) and three untreated control lambs (*n* = 7 isolates) and conducted a broth microdilution assay to test the *in vitro* susceptibility to enrofloxacin (Figure 5). The isolates showed variability in the minimum inhibitory concentration (MIC) ranging from 0.005 to 8.1 μg/mL. Interestingly, one out of five isolates from lamb (8216DB) with re-emergent *M. ovipneumoniae* infection following enrofloxacin treatment had a high MIC value of 20 ± 0.00 μg/mL with transfer (passage) T10, in two independent experiments (Figures 5A, B). However, the MIC values from the same isolate kept frozen for a year and analyzed at a transfer (passage) T12 was 0.134 ± 0.039 μg/mL (mean ± SD) in three additional experiments. In contrast, MICs for the isolates obtained from the untreated control lambs 7022DB, 7414DB, and 8020DB ranged from 0.010 to 0.055 μg/mL. As a reference, the MIC of *M. ovipneumoniae* type strain Y98 was measured as 0.004 ± 0.001 μg/mL (mean ± SD; Figure 5B).

**Figure 5:**
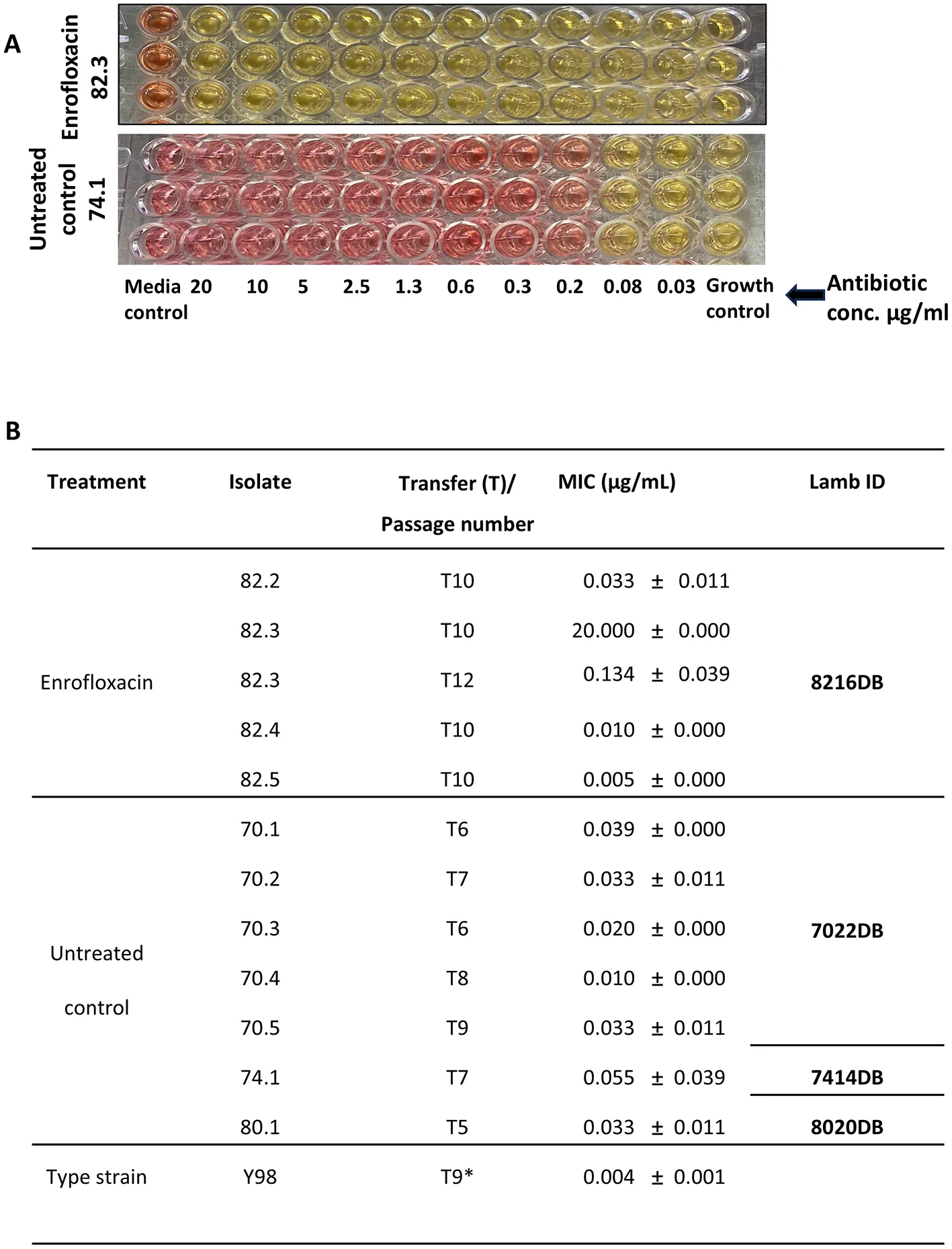
*M. ovipneumoniae* isolates recovered from lung tissue show variable susceptibility to enrofloxacin. **(A)** Representative images of two *M. ovipneumoniae* culture plates showing color change from red to yellow indicating growth of the bacteria with different concentrations of enrofloxacin. **(B)** Minimum inhibitory concentration (MIC) for different *M. ovipneumoniae* isolates cultivated from frozen lung tissue samples. MIC values are expressed as mean ± SD from three technical replicates for each isolate. For isolate 82.3 T10, mean ± SD represents two independent experiments; for 82.3 T12, mean ± SD represents three independent experiments. *For Y98, transfer number reflects number of transfers performed after receiving the strain from ATCC.

Similarly, the five *M. haemolytica* isolates had variable MIC values from 0.001 to 0.25 μg/mL. Two isolates were recovered from the same lamb (8216DB) with reemerging *M. haemolytica* infection before and after enrofloxacin treatment. Both isolates (MH82.1 and MH82.2) had an MIC of 0.25 μg/mL, whereas the Newsom and Cross type strain of *M. haemolytica* had an MIC of 0.078 μg/mL. Notably, all MICs for determined for *M. haemolytica* were considered to be in the susceptible range based on the CLSI guidelines (31) (Figures 6A, B).

**Figure 6:**
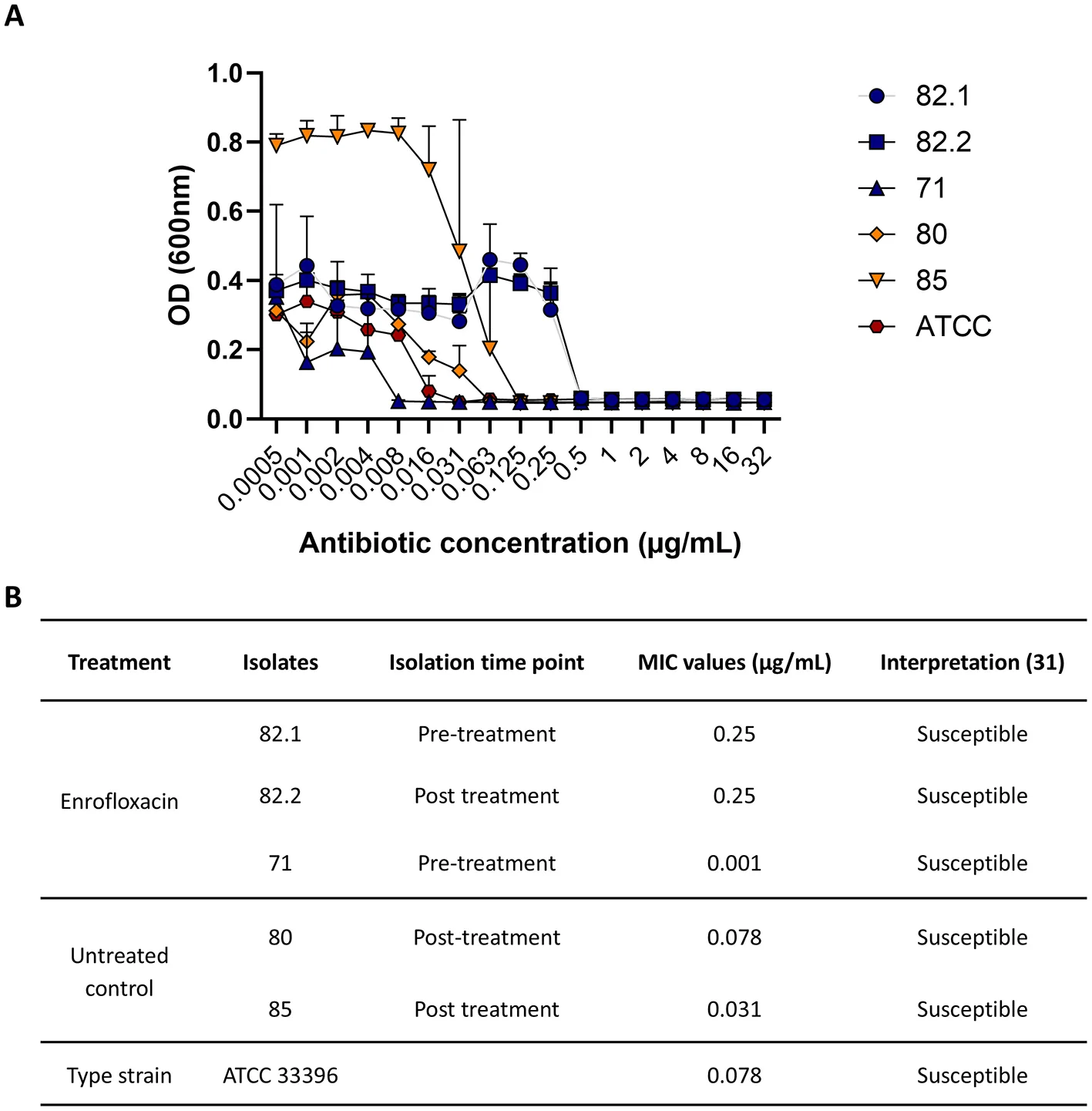
*M. haemolytica* isolates from nasal swabs show variable susceptibility to enrofloxacin. **(A)** Graph representing the optical density of five *M. haemolytica* isolates (all at passage 4) and the type strain ATCC 33396 indicating growth of the bacteria in that antibiotic concentration incubated overnight in an incubator shaker with an initial OD600 of < 0.05 for all isolates. Graph shows mean ± SD of the OD reading of the three technical replicates of each isolate. **(B)** Minimum inhibitory concentrations (MIC) of the *M. haemolytica* isolates detected from the OD values shown in panel **(A)** with interpretation of the test results.

### 3.7 Antibiotic treatment leads to no significant improvement in health scores and daily weight gains in the treated lambs

We measured the daily health scores, biweekly body temperatures, and average daily weight gains based on weekly body weights in both lamb groups throughout the study period to evaluate whether enrofloxacin treatment of *M. ovipneumoniae* infection led to improved lamb productivity. In general, no major clinical signs of respiratory disease were observed beyond occasional sneezing and elevated body temperatures (Figure 7A). Health scores trended lower in the enrofloxacin treated lambs (Figure 7A). Body temperatures also trended lower for the enrofloxacin-treated animals (Figure 7B), but weight gains were similar in the two groups (Figure 7C).

**Figure 7:**
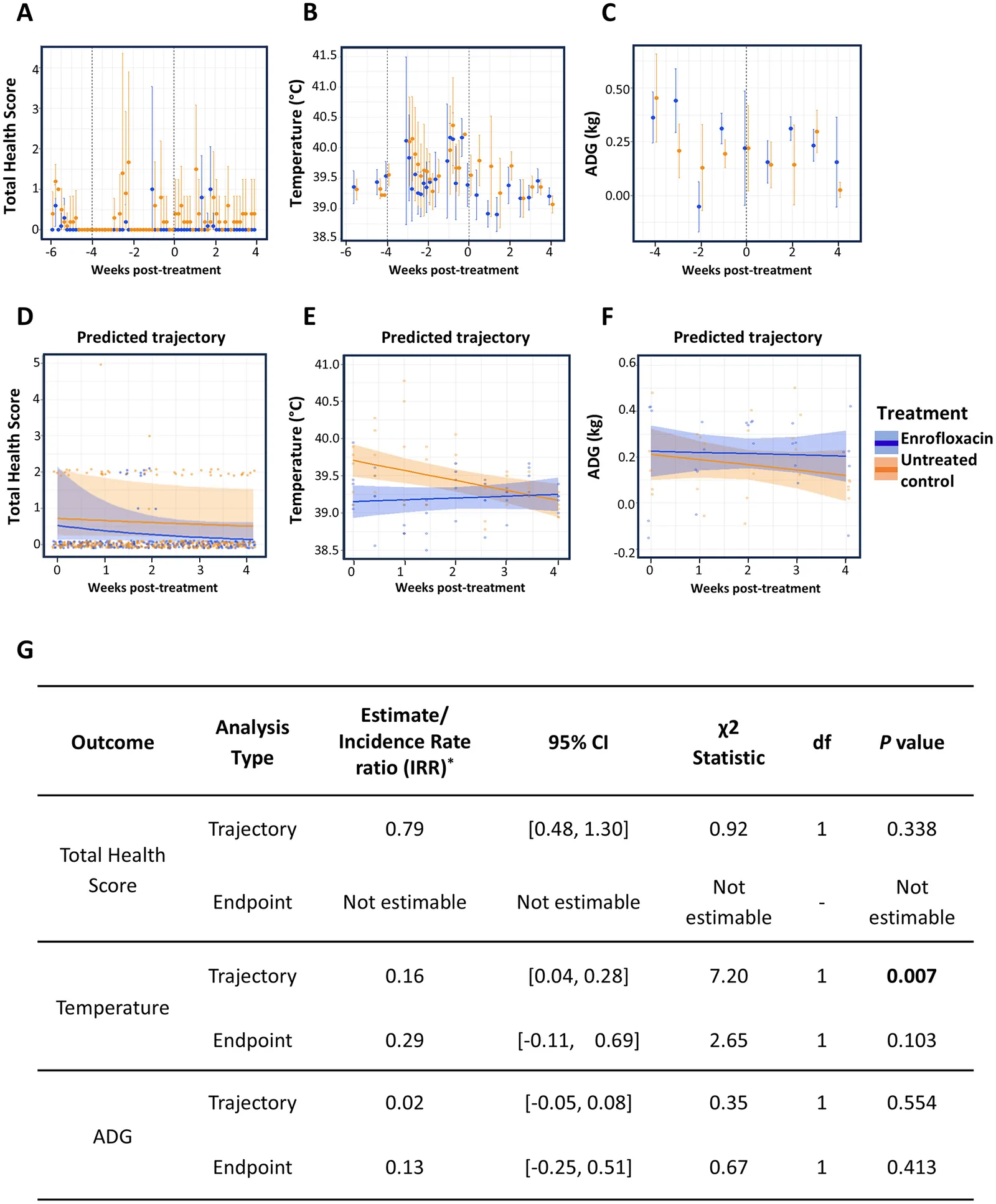
Enrofloxacin treatment resulted in no major differences in the total health scores, body temperature, or average daily gains in lambs infected with *M. ovipneumoniae*. **(A)** Total health scores calculated from all lambs over 10 weeks, from 2 weeks prior to *M. ovipneumoniae* infection to 4 weeks after treatment based on twice daily scoring of behavior, appetite, respiratory signs, and administered medication. **(B)** Body temperature measured twice weekly over 10 weeks from 2 weeks prior to *M. ovipneumoniae* infection to 4 weeks after treatment. **(C)** Average daily weight gain (ADG) measured weekly for 8 weeks from infection to 4 weeks after treatment. The ADG at week-4 was calculated based on the body weights measured at week-6. For panels **(A–C)**, the gray dotted lines denote the infection (−4 weeks) and treatment (0 weeks) time points. All graph in these panels show mean ± SD of *n* = 5 lambs per group. **(D–F)** Model-predicted trajectories showing 95% confidence intervals for (**D**) the total health score data observed in the post-treatment period, calculated using a zero-inflated Poisson generalized linear mixed model; **(E)** biweekly body temperatures observed in the post-treatment period, calculated using a linear mixed model; **(F)** ADGs observed in the post-treatment period, calculated using a linear mixed model. For panels **(A–F)**, blue lines correspond to enrofloxacin-treated lambs, and orange lines to untreated controls. **(G)** Summary table of the estimated effect sizes, 95% confidence intervals, and Likelihood Ratio Test statistics for the trajectory and endpoint mixed-effects models across all three clinical outcomes. Health score counts for the enrofloxacin-treated group dropped to zero in week 4, resulting in complete separation of the data. Consequently, valid model estimates, confidence intervals, and Likelihood Ratio Test statistics could not be computed for this endpoint. *Estimates are reported as incidence rate ratio (IRR) for total health score and as linear estimates for temperature and ADG.

A linear trajectory model was fit to assess lamb health over the 4-week post-treatment period (Figures 7D–G). While visual trends suggested that the enrofloxacin group achieved lower (healthier) scores faster than the untreated control group, the Treatment × Time interaction was not statistically significant [Incidence rate ratio = 0.79, 95% CI = (0.48, 1.30), $\chi_{\left(1\right)}^{2}$ = 0.92, *P* = 0.338], likely due to the limited sample size (*n* = 5 per group) reducing statistical power. An endpoint analysis was performed to compare final health scores between the two groups after 4 weeks. Visually, the treated group trended toward a complete resolution of clinical signs, with health score counts dropping to zero at the study endpoint. However, this caused complete separation in the data. Consequently, valid model estimates and Likelihood Ratio Test statistics could not be computed to formally confirm this trend. In analyzing the rate of temperature decline, the longitudinal trajectory analysis showed a significant Time and Treatment interaction with body temperature [estimate = 0.16, 95% CI = (0.04, 0.28), $\chi_{\left(1\right)}^{2}$ =7.20, *P* = 0.007], indicating that antibiotic administration modified the rate of temperature decline after treatment in the lambs. However, the endpoint analysis did not find a significant interaction between week 0 and week 4 [estimate = 0.29, 95% CI = [−0.11, 0.69], $\chi_{\left(1\right)}^{2}$ = 2.65, *P* = 0.103]. Furthermore, in analyzing ADG, no significant Treatment × Time interaction was observed in the longitudinal trajectory analysis [estimate = 0.02, 95% CI = (−0.05, 0.08), $\chi_{\left(1\right)}^{2}$ = 0.35, *P* = 0.554] and the endpoint analysis for ADG between Week 0 and Week 4 [estimate = 0.13, 95% CI = (−0.25, 0.51), $\chi_{\left(1\right)}^{2}$ = 0.67, *P* = 0.413]. Collectively, these results suggest that the treatment and control groups achieved statistically similar outcomes. By the end of the study, both the enrofloxacin and control groups demonstrated no statistically significant differences in clinical signs or average daily gain.

## 4 Discussion

In this pilot study, we used an upper respiratory tract infection model in a small group of specific pathogen-free lambs to determine whether local and systemic enrofloxacin (Baytril^®^) treatment could clear or ameliorate *M. ovipneumoniae* infection in domestic lambs. Enrofloxacin has been used for the treatment and prevention of respiratory infections with *Mycoplasma spp*. in livestock and poultry (38–40). It has a broad-spectrum antimicrobial activity, acting as bactericidal agent against gram-positive and gram-negative bacteria and *Mycoplasma spp*. by inhibiting bacterial gyrase and topoisomerase IV enzymatic actions (41, 42). Due to the absence of cell walls, *Mycoplasma spp*. is highly resistant to multiple commonly used classes of antibiotics such as beta-lactams and glycopeptides that target bacterial cell wall synthesis (43, 44). Enrofloxacin for treatment of *M. bovis* infection in cattle resulted in reduced clinical and lung lesions (39). *M. hyopneumoniae-*infected swine showed significant improvement in average weight gain, feed intake, and feed efficiency in response to enrofloxacin treatment (45). *M. ovipneumoniae* was shown to be susceptible to a wide range of fluoroquinolones, macrolides and tetracycline *in vitro* (21, 22, 24, 46). However, oxytetracycline, lincomycin, florfenicol, and gamithromycin had no significant efficacy against *M. ovipneumoniae* infection in domestic sheep *in vivo* (24, 47). Therefore, we used enrofloxacin (Baytril^®^) to determine its therapeutic efficiency against *M. ovipneumoniae* infection in domestic lambs.

Our study showed that a 5-day course of intranasal and systemic enrofloxacin led to a partial clearance of *M. ovipneumoniae* from the nasal cavity. Based on nasal swab PCR analysis, all five treated lambs tested *M. ovipneumoniae-*negative at 1 week after initiation of treatment. While two of the treated lambs remained *M. ovipneumoniae-*negative through the end of the experiment at 4 weeks post treatment, the infection reemerged in the nasal cavity 2–3 weeks after treatment in 3/5 (60%) of the enrofloxacin-treated lambs. These findings confirm recently published data by Magnusson et al. (48), where the prevalence of *M. ovipneumoniae* positive sheep in a naturally infected domestic flock decreased from 90% to 23% at day five post enrofloxacin treatment and then rebounded to 86% at 5 weeks post enrofloxacin treatment. Overall, our data showed that the intranasal and subcutaneous administration of enrofloxacin has a positive, but incomplete therapeutic effect against nasal colonization with *M. ovipneumoniae* in domestic sheep.

The reemergence of *M. ovipneumoniae* infection in the nasal cavity could be caused by incomplete elimination of *M. ovipneumoniae* from the lower respiratory tract or other anatomical niches by the treatment. In humans, several respiratory tract infections are associated with inflammation of middle ear termed otitis media (49–51). Besser et al. (18) has demonstrated the presence of purulent otitis media in three *M. ovipneumoniae* infected bighorn sheep postmortem, and this is commonly seen in infected young bighorn lambs. Indeed, using PCR testing, we demonstrated the persistence of *M. ovipneumoniae* in the middle ear of all enrofloxacin-treated lambs in our study, pointing to the middle ear as a site of persistent colonization. As the middle ear is surrounded by a tissue barrier composed of bone, antibiotic penetration into the middle ear is typically low (52), which may have caused the treatment failure in our study. However, in humans, antibiotic concentrations above the MIC can be achieved in the middle ear and inhibit bacterial growth (53), and successful topical antibiotic treatment of the tympanic membrane has also been reported (54). Possibly, improved antibiotic delivery to the middle ear may be necessary to achieve antimicrobial treatment success in ovine *M. ovipneumoniae* infection but will require additional research efforts.

Alternatively, reemergence of the infection could be due to the expansion or evolution of enrofloxacin-resistant *M. ovipneumoniae*. Mycoplasmas may acquire fluoroquinolone resistance due to mutations in the DNA gyrase *gyrA* (55). In support of this alternative hypothesis, Gaeta et al. (56) found *gyrA* mutations in multiple *M. ovipneumoniae* strains. To investigate whether the recurrent *M. ovipneumoniae* infection in the enrofloxacin-treated lambs was due to antimicrobial resistance, we recovered eleven *M. ovipneumoniae* isolates from the lungs of infected lambs and performed a broth microdilution antibiotic susceptibility assay with enrofloxacin. Using the guidelines for determining the minimum inhibitory concentration (MIC) for Mycoplasma species proposed by Hannan (30), we found variable MIC values for the different isolates. One out of four isolates (82.3) from lamb 8216DB, which had recurrent *M. ovipneumoniae* infection after enrofloxacin treatment, had a high MIC value (20 μg/mL) at transfer #10. However, the other three isolates from the same lamb had lower MIC values ranging from 0.005 to 0.033 μg/mL. Interestingly, the MIC value for isolate 82.3 dropped to 0.134 at transfer #12, which may have been caused by genetic or epigenetic changes during *ex vivo* culture. Similarly, the five isolates from the same untreated control lamb 7022DB and two isolated from other lambs 7414DB and 8020DB had variable MIC values ranging from 0.010 to 0.055 μg/mL. The type strain Y98 *M. ovipneumoniae* strain had the lowest MIC value at 0.004 ± 0.001 μg/mL. These results were similar to Jay et al. (21), who used an agar dilution method for 28 ovine *M. ovipneumoniae* isolates and found variable MIC values against enrofloxacin ranging from 0.0625 to 0.25 μg/mL Since no clinical interpretation criteria for the MIC values of *M. ovipneumoniae* are available (57), antimicrobial resistance in these isolates cannot be confirmed. Our data showing that an *M. ovipneumoniae* isolate recovered from an enrofloxacin treated lamb with recurrent infection had a higher MIC value than an *M. ovipneumoniae* isolate from an untreated control lamb support the hypothesis that the *M. ovipneumoniae* either developed antimicrobial resistance in response to the treatment, or that a resistant isolate that may have already been present in the mixed inoculum administered to the lambs was able to expand upon enrofloxacin exposure.

Interestingly, we also observed re-infection with *M. haemolytica* in the sample lambs that showed re-infection with *M. ovipneumoniae. M. haemolytica* isolates from the nasal cavities of two enrofloxacin-treated and two untreated lambs had variable MIC values for enrofloxacin ranging from 0.001 to 0.25 μg/mL. Interestingly, the highest MIC (0.25 μg/mL) was detected in two *M. haemolytica* isolates collected before and after enrofloxacin treatment from one lamb with recurrent infection. This suggests that this lamb was infected with a less susceptible *M. haemolytica* strain before the treatment, but there was no development of antibiotic resistance. However, whether any of the lambs harbored enrofloxacin-resistant *M. ovipneumoniae* strains before the treatment remains unknown, as isolation of culturable *M. ovipneumoniae* from nasal swabs was not feasible.

Our study design did not enable us to determine whether lambs with recurrent infection were re-infected by other animals in the group or whether they continuously harbored *M. ovipneumoniae* at levels below the detection limit or at sites such as the middle ear that were inaccessible for routine sampling. Due to the setup of our large animal BSL2-facility, which is not equipped for individual animal housing, all lambs from one experimental group were comingled during the entire experiment, potentially enabling *M. ovipneumoniae* transmission. Reinfection of the lambs after successful treatment from environmental sources in the animal room also cannot be excluded but is unlikely due to daily cleaning and disinfection protocols. A study design where animals would be housed in individual rooms, or, alternatively, detailed whole genome sequencing and genetic comparison of *M. ovipneumoniae* isolates recovered from each animal before and after treatment, could help differentiate between re-infection vs. continuous infection.

This is the first study, to our knowledge, to analyze the effectiveness of enrofloxacin treatment to ameliorate *M. ovipneumoniae*-associated clinical disease and respiratory pathology in a small group of animals. As described in the introduction, *M. ovipneumoniae* infection in sheep is associated with histopathological lesions of the respiratory tract, clinical respiratory illness, reduced average daily weight gains (ADG) and lamb productivity (13, 58). Our study showed histopathological alterations in the lungs of some of the lambs from both groups that included BALT hyperplasia, peribronchiolar lymphocyte accumulation, and neutrophil infiltration of the alveoli, which are consistent with previous reports on *M. ovipneumoniae* infection (46, 59). Buddle et al. (60) described mild peri-bronchial lymphoid hyperplasia and alveolar interstitial infiltrates after experimental *M. ovipneumoniae* and secondary *M. haemolytica* infection. In our study, there was a general trend for lower histopathological lung scores in the treated lambs, indicative of therapeutic effects of the antibiotic treatment. In cattle, a study using enrofloxacin (Enflocyna^®^, Biowet) at the dose of 5 mg/kg body weight once a day for 3 days showed reduced clinical scores with *M. bovis* infection (39). In pigs, use of enrofloxacin as a premix formula in feed resulted in significant improvement in daily weight gain for the treatment of *M. hyopneumoniae* (45). In contrast, no such effects were detected in our study, with no significant changes in health scores, body temperatures, or ADG in the enrofloxacin-treated compared to the untreated group. This lack of improvement in these clinical parameters could be due to the relatively mild clinical signs observed in both groups of lambs throughout the study, or the predominance of low virulence *M. ovipneumoniae* isolates in the mixed nasal wash inocula that were used in our study. While the analyses suggested a trend toward faster recovery in the treated group, enrofloxacin did not significantly alter the final clinical outcomes. This is due to the relatively small sample size (*n* = 5 per group) which led to data sparsity that restricted our statistical power to definitively confirm this finding or establish long-term outcomes. Therefore, a larger sample size would be required in future studies to statistically confirm the altered rate of improvement. Repeating this pilot-scale study with a higher number of animals might also reveal significant effects of enrofloxacin treatment on weight gains or respiratory pathology.

As mentioned, primary infection with *M. ovipneumoniae* facilitates secondary bacterial infection with *Pasteurellaceae* causing polymicrobial pneumonia in domestic and wild sheep (61, 62). In our study, we infected SPF lambs with nasal wash samples from sheep naturally infected with *M. ovipneumoniae*. The nasal wash samples were treated with ceftiofur, and standard microbiological analysis showed that the ceftiofur treatment successfully eliminated viable bacteria from the inoculum. Given that the SPF flock has consistently tested negative for *M. haemolytica* over 6 years, the detection of *M. haemolytica* infection in the lambs was unexpected. We hypothesize that this infection was caused by *M. haemolytica* that remained in the nasal wash inocula after ceftiofur treatment and that was below the detection limit of the microbiological assays. While this hypothesis cannot be confirmed, the emergence of *M. haemolytica* highlights the unique ability of *M. ovipneumoniae* to enable the expansion of secondary bacterial pathogens in the ovine respiratory tract. Interestingly, the same three lambs that tested *M. ovipneumoniae* positive in the enrofloxacin treated group also were *M. haemolytica* positive four weeks post treatment. The significantly lower *M. haemolytica* colonization of the caudal nasal cavity in the enrofloxacin-treated lambs compared to the untreated controls suggests successful suppression of *M. haemolytica* infection by enrofloxacin.

## 5 Conclusion

In this study, we evaluated the treatment efficacy of the antibiotic enrofloxacin in 3-month-old lambs experimentally infected with *M. ovipneumoniae*. This treatment was assessed using qPCR for pathogen detection in the nasal cavity, paranasal sinus, bronchi, trachea, recording clinical signs associated with this bacterial infection, examining the postmortem gross and histological lesions, and assessing the presence of any secondary bacterial infection. Enrofloxacin treatment resulted in short-term eradication of *M. ovipneumoniae* infection from the nasal cavity, which was consistent with the high *in vitro* susceptibility observed for the reference strain. However, the re-emergence of infection in a subset of treated animals over the post-treatment period indicates that enrofloxacin alone was insufficient to achieve sustained clearance. There are multiple hypotheses for the reemergence such as development of antimicrobial resistance in the pathogens or inoculation of the lambs with resistant strains of *M. ovipneumoniae*. The detection of the pathogen in the middle ear of all infected lambs points to potential *M. ovipneumoniae* reservoirs outside the respiratory tract. Similarly, the ability of biofilm formation by various *Mycoplasma* species could be another potential reason for antimicrobial resistance and hence the reemergence. Overall, our findings highlight the ability of *M. ovipneumoniae* to persist despite antimicrobial treatment and reflects the limitations of antibiotic treatment for *M. ovipneumoniae* eradication in domestic lambs.

## Data Availability

The original contributions presented in the study are included in the article/Supplementary material including the source data (Supplementary File 3), further inquiries can be directed to the corresponding author.
