# Supplementary material for: Limited efficacy of enrofloxacin against Mesomycoplasma ovipneumoniae infection in domestic lambs
Source: Front Vet Sci. 2026 Oct 22;13:1944866. doi: 10.3389/fvets.2026.1944866 (PMC13736132; doi:10.3389/fvets.2026.1944866)
Supplement: Supplementary file 1 [file Supplementary_File_1.pdf]

M. ovipneumoniae PCR standard curve calculation

- 1.6^6 cells/mL bacteria was diluted seven times up to concentration of 1.6^0 cells/mL.
- DNA were extracted from this diluted samples and PCR was performed in replicates of five.

Dilution	Ct values					Cell count/ml
0	15.967	15.67	15.51	15.46	15.526	1.6^6
-1	20.499	20.394	19.695	19.683	19.75	1.6^5
-2	24.878	24.672	23.841	23.962	23.965	1.6^4
-3	28.09	28.318	27.802	27.514	27.65	1.6^3
-4	32.768	32.556	32.075	31.8	32.625	1.6^2
-5	35.038	35.244	35.196	34.301	34.987	1.6^1
-6	40	37.881	40	40	38.129	1.6^0

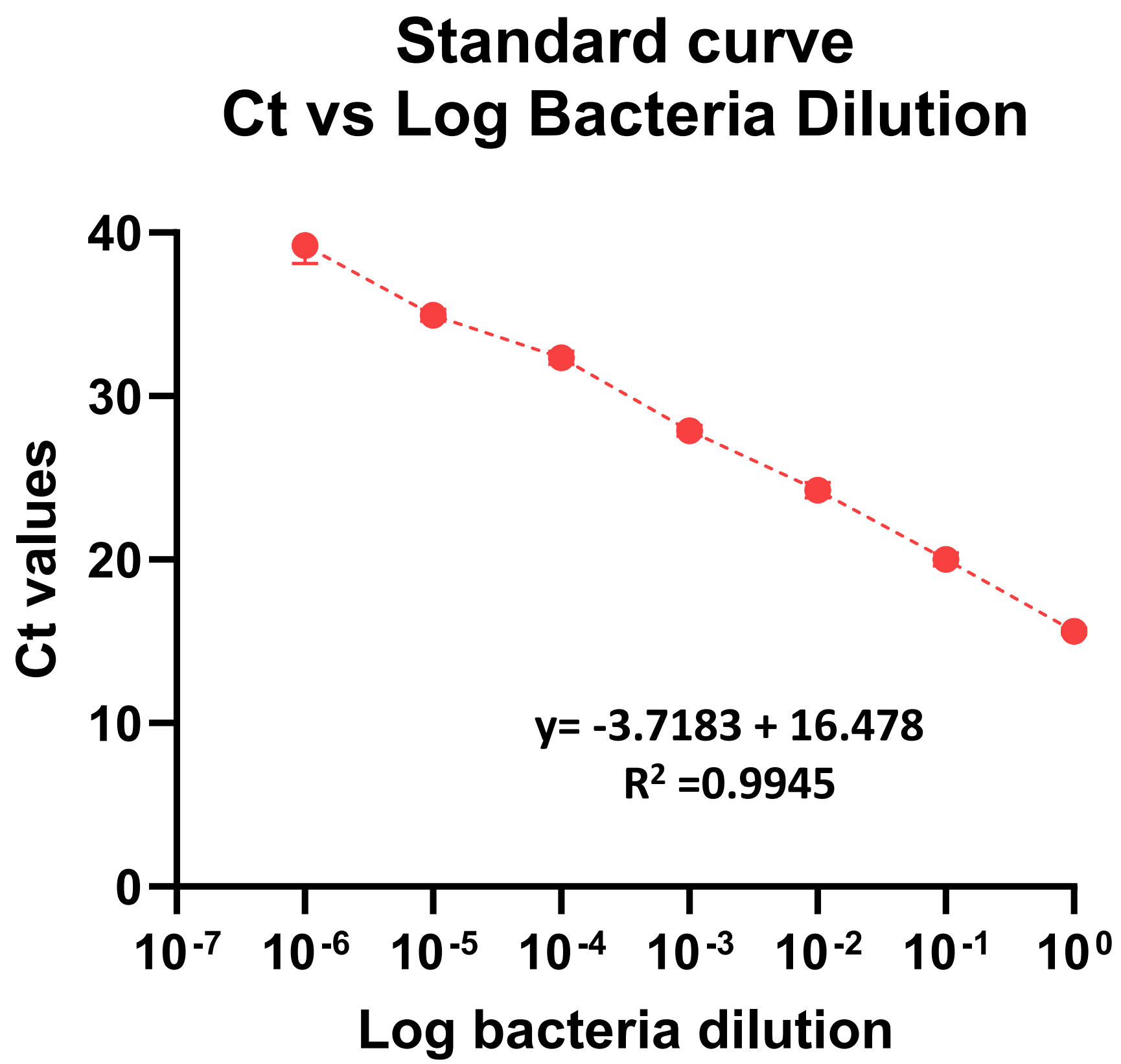

Calculation for PCR efficiency

E = {10^(–1/S)} – 1 (where S = slope of the standard curve)

E= {10^(–1/-3.7183)} – 1

E= 85.8%

M. haemolytica PCR standard curve calculation

- 10^6 CFUs/mL bacteria was diluted seven times up to concentration of 10^0 CFUs/mL.
- DNA were extracted from this diluted samples and PCR was performed in replicates of five.

Dilution	Ct values					CFUs/ml
0	14.228	14.408	14.084	14.052	13.772	10^6
-1	17.26	17.568	17.376	17.407	17.313	10^5
-2	20.825	20.821	20.606	20.771	20.323	10^4
-3	23.896	23.983	24.009	23.817	23.816	10^3
-4	27.675	27.64	27.369	27.402	27.711	10^2
-5	30.069	30.535	30.083	30.429	30.059	10^1
-6	35.381	35.319	35.26	35.351	35.291	10^0

Standard curve
Ct vs Log Bacteria Dilution

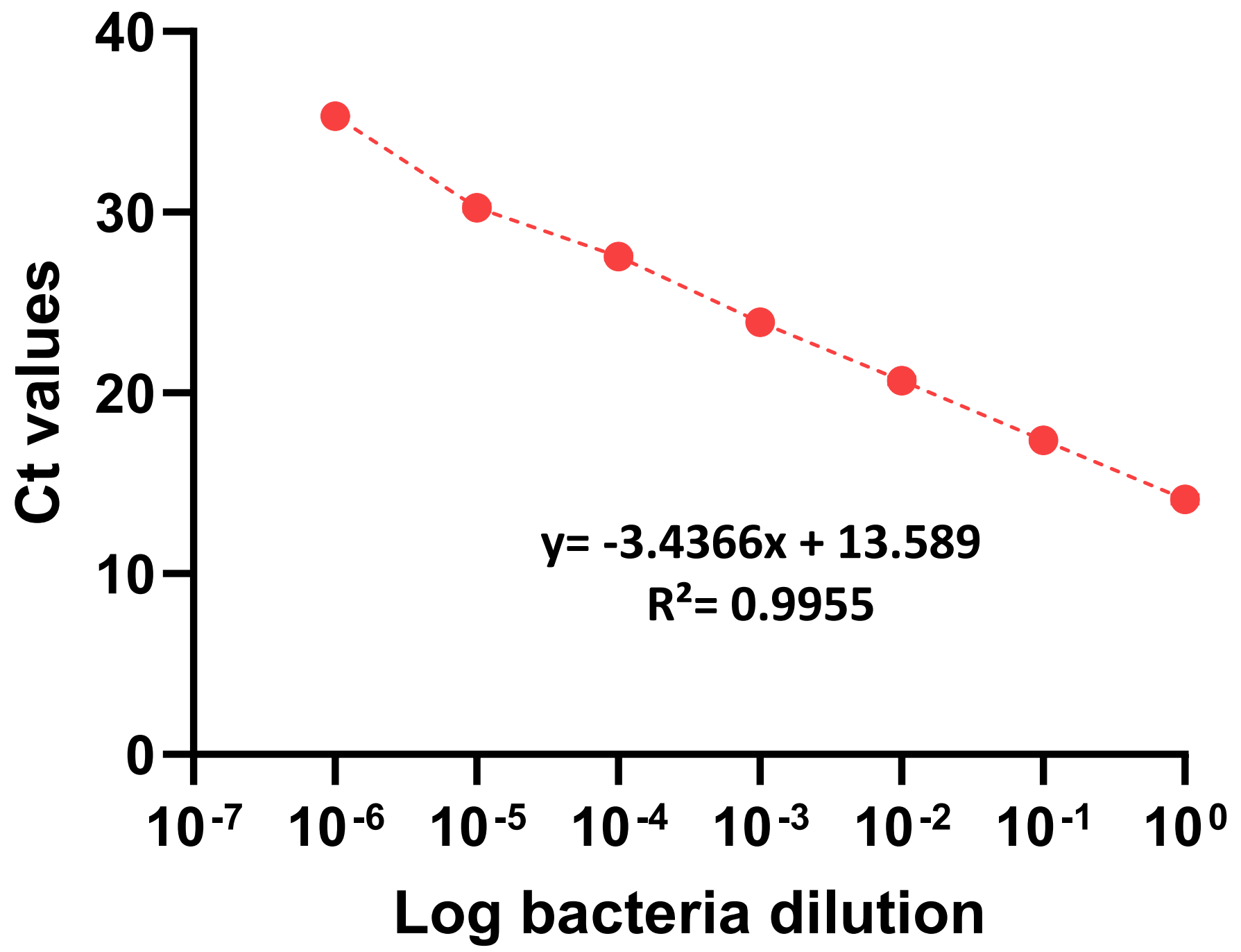

Calculation for PCR efficiency

E = {10^(–1/S)} – 1 (where S = slope of the standard curve)

E= {10^(–1/-3.4366)} – 1

E= 95.4%
