# Supplementary material for: Limited efficacy of enrofloxacin against Mesomycoplasma ovipneumoniae infection in domestic lambs
Source: Front Vet Sci. 2026 Oct 22;13:1944866. doi: 10.3389/fvets.2026.1944866 (PMC13736132; doi:10.3389/fvets.2026.1944866)
Supplement: Supplementary file 2 [file Supplementary_File_3.pdf]

Supplementary Material

1 Supplementary Tables

Supplementary Table 1: Overview of the lambs and key experimental findings per animal

	**Enrofloxacin**					**Untreated control**				
	**7122DB**	**7222DB**	**7625DB**	**7925DB**	**8216DB**	**7022DB**	**7414DB**	**8020DB**	**8116DB**	**8518DB**
**Histo-pathology**	Conges-tion	No lesions	Conges-tion	No lesions	Edema and conges-tion	Edema and BALT	Interstitial inflammation, hemorrhage, congestion	Edema	Inter-stitial inflamm-ation, conges-tion, BALT	No lesions
**Gross pathology**	No lesions	No lesions	Enlarged lymph node	Fibrous adhesions	Hemor-rhagic	Mildly enlarged lymph node	Consolidation and pleural adhesions	No lesions	Consoli-dation	No lesions
**TEM**	Intact cilia	ND	ND	Intact cilia	ND	ND	ND	ND	Ciliary damage	Ciliary damage
***M. haem* PCR 4 weeks post treatment**	positive	negative	positive	negative	positive	positive	positive	positive	positive	positive
***M. ovi* PCR 4 weeks post treatment**	positive	negative	positive	negative	positive	positive	positive	positive	positive	positive

Supplementary Table 2: Health scoring parameters for the lambs during the experimental period (1)

Parameters	0	1	2	3	4	5
**Behavior**	Bright, alert, responsive	Small change in attitude/ behavior	Less active, visibly appears ill	Ill but still responsive	Lethargic	Unresponsive
**Appetite**	Ate well	Ate most of the ration	Ate ¾ feed	Ate ½ feed	Ate < ½ feed	Not eating
**Respiratory Signs**	No nasal or ocular discharge, no cough, normal respiration	Panting/ increased respiratory rate, or slight clear nasal/ocular discharge	Clear nasal or ocular discharge, or coughed 1-5 times	Moderate ocular or nasal discharge or frequent coughing	Mucous, nasal, or ocular discharge, coughing, lamb visibly unwell	Discharge, coughing, wheezing, labored breathing
**Medication**	No treatments	Electrolytes	NSAID	Antibiotic	NSAID and antibiotics	Multiple NSAIDs and antibiotics

Supplementary Table 3: Composition of *Mycoplasma* media

Reagent	Manufacturer/Catalog	Concentration
Mycoplasma broth	Fisher/ B11458	22.4 g/L
Dextrose anhydrous	Fisher/ BP350500	3 g/L
L-cysteine hydrochloride	Fisher/ BP376-100	0.1 g/L
Beta-NAD	Fisher/ 501800403	0.1 g/L
Thallium acetate	Sigma Aldrich/ T8266	0.5 g/L
Phenol red	Sigma Aldrich/ P3532	0.024 g/L
Porcine serum	Fisher/ 26-250-084	75 mL/L
Horse serum	Fisher/ 16-050-122	75 mL/L
Penicillin G	Fisher/ MP219453625	0.52 g/L

1. Jacobson BT, DeWit-Dibbert J, Zanca L, Sonar S, Hardy C, Throolin M, et al. Pathogen delivery route impacts disease severity in experimental Mycoplasma ovipneumoniae infection of domestic lambs. Vet Res. 2025;56(1):10.
